# Artificial Intelligence in Colorectal Cancer Diagnosis Using Clinical Data: Non-Invasive Approach

**DOI:** 10.3390/diagnostics11030514

**Published:** 2021-03-14

**Authors:** Noémi Lorenzovici, Eva-H. Dulf, Teodora Mocan, Lucian Mocan

**Affiliations:** 1 Department of Automation, Faculty of Automation and Computer Science, Technical University of Cluj-Napoca, Memorandumului Str. 28, 400014 Cluj-Napoca, Romania; Noemi-Ordogh@student.utcluj.ro; 2 Physiological Controls Research Center, Óbuda University, H-1034 Budapest, Hungary; 3 Department of Physiology, Iuliu Hatieganu University of Medicine and Pharmacy, 400000 Cluj-Napoca, Romania; teodora.mocan@umfcluj.ro; 4 Nanomedicine Department, Regional Institute of Gatroenterology and Hepatology, 400162 Cluj-Napoca, Romania; 5 Department of Surgery, 3-rd Surgery Clinic, Iuliu Hatieganu University of Medicine and Pharmacy, 400000 Cluj-Napoca, Romania; lucian.mocan@umfcluj.ro

**Keywords:** artificial intelligence, deep neural networks, colorectal cancer, clinical data, computer aided diagnostic system

## Abstract

Colorectal cancer is the third most common and second most lethal tumor globally, causing 900,000 deaths annually. In this research, a computer aided diagnosis system was designed that detects colorectal cancer, using an innovative dataset composing of both numeric (blood and urine analysis) and qualitative data (living environment of the patient, tumor position, T, N, M, Dukes classification, associated pathology, technical approach, complications, incidents, ultrasonography-dimensions as well as localization). The intelligent computer aided colorectal cancer diagnosis system was designed using different machine learning techniques, such as classification and shallow and deep neural networks. The maximum accuracy obtained from solving the binary classification problem with traditional machine learning algorithms was 77.8%. However, the regression problem solved with deep neural networks yielded with significantly better performance in terms of mean squared error minimization, reaching the value of 0.0000529.

## 1. Introduction

Colorectal cancer (CRC) is the third most common and second most lethal tumor globally, causing 900,000 deaths annually. According to the International Agency for research on Cancer in 2018, 1.8 million people were newly diagnosed with colorectal cancer that year [1]. Additionally, it is predicted that by the year 2030, the prevalence of colorectal cancer worldwide is going to increase by approximately 60%, in terms of diagnosing 2.2 million new cases annually and causing more than 1.1 million deaths. Additionally, the chances of developing other cancer types after being diagnosed with colorectal cancer are significantly high: colorectal cancer is the third major cause of lung and liver cancers [2,3].

The two fundamental steps in tumor recognition are: evaluation of some categorical and numeric data (typically obtained from a blood test), as well as the inspection of a medical image taken of the patient. There are a wide variety of medical imaging technologies, the most frequent of them being MRI, ultrasound, CT scan, endoscopy and X-ray [4]. The most common technique used nowadays for colorectal tumor detection and screening is colonoscopy [5]. Although widely used, colonoscopy has a tumor miss-rate as high as 24% leading to medical malpractice [5]. Furthermore, performing a comprehensive colonoscopy of an individual can take from 10 min to several hours while making hundreds, sometimes even thousands of frames. Since not all these frames offer the gastroenterologist useful information, the examination of them is a demanding and elongated task, making the odds of missing them even higher [6]. Research has proved that if endoscopic diagnose adenoma during colonoscopy, their patient’s risk of having that adenoma turn into colonic cancer reduces significantly. A study even demonstrated that every 1% raise in polyp detection leads to a 3% decrease in the risk of colonic cancer, thus making the importance of developing an automatic polyp-recognition system even more urgent [7].

Obtaining a precise diagnosis in the first phase of the medical examination, from clinical data, can reduce both the risk of human error and save time so that experts can make colonoscopies only for patients at high risk of having cancer. Such data can be obtained from the blood, saliva and fecal of the patient. At the moment there are two major types of blood data used in cancer diagnosis: genomic and proteomic. Genomic data can be epigenetic, circulating tumor DNA, MicroRNAs and stool-based tests [8].

A recent study [9] attempted to identify stomach and colorectal cancer through analyzing the following oxygen-containing salivary volatile organic compounds (VOCs): acetaldehyde, acetone, propanol-2 and ethanol. In order to attain the diagnosis, classification and regression trees were used. According to the paper, the results were promising: the constructed diagnosis system had a sensitivity equal to 95.7% and a specificity of 90.9%.

Numerous papers in an emerging research field inspect the efficiency of blood biomarkers in the early detection of cancer [10]. A work [11] on this matter points out that there is only one biomarker that is an internationally approved clinical practice for colorectal cancer detection: carcinoembryonic antigen (CAE). In addition, by comparing healthy tissues with carcinogen ones and performing protein expression analysis on them, the study offers a novel method to search for other possible biomarkers in order to detect colorectal cancer. 

Recently, the CAE antigen has been compared to other novel serum biomarkers with the hope of finding a more reliable CRC diagnosis method. One approach consisted of inspecting the accuracy of a plasma biomarker, Alpha 1-antitrypsin (A1AT) activity and comparing it with the accuracy of CAE. The serum A1AT activity proved to be a more efficient biomarker than the CAE, with a higher sensitivity and specificity. Moreover, the study concluded that the concentration of A1AT in the blood indicates the phases of the tumor growth in a well-distinguishable way [12]. 

The novel combination of a set of clinical data presented in this study was inspired also by the scientific work [13] published in 2019. The mentioned paper analyzes a novel sequence of plasma biomarkers, namely the following circulating lncRNAs: ZFAS1, SNHG11, LINC00909 and LINC00654 to diagnose CRC. Along with promising results, according to which these biomarkers help in CRC detection, the paper also demonstrates the significance of an exact combination of biomarkers and states that a more abundant set of biomarkers might provide with even better results. 

The efficiency of circular RNAs in colorectal cancer discovery is inspected in many research papers. One of them reveals the plasma of patients with CRC contains a significantly reduced amount of the following three circular RNAs: circ-CCDC66, circ-ABCC1 and circ-STIL. For further development, the study also points out that combining these three biomarkers with the widely used CAE marker could improve the early diagnosis of CRC [14].

Even after successful treatment the chance of developing recurrent colorectal cancer is 30–50%. Another approach that is gaining popularity in diagnosing recurrent CRC involves the inspection of epigenetic biomarkers, such as cell-free circulating tumor DNA (ctDNA). Excessive gene methylation is a frequent phenomenon in colorectal cancer, which can be observed in ctDNA as well [15,16]. Additionally, after comparing this innovative biomarker with the carcinoembryonic antigen in study [17], the ctDNA proved to be twice as sensitive as the CEA. 

Similarly, the observational study [18] on genetic biomarkers compared a novel 2-gene dataset (methylated BCAT1 and IKZF1) with CEA and concluded that the set of 2-gene biomarkers yielded significantly higher sensitivity for diagnosing recurrent colorectal cancer than the CEA biomarker.

On the other hand, the role of clinical data in colorectal cancer treatment exceeds early diagnosis: they can also offer a new alternative way of treatment. For example, research [19] analyzes the immunoglobulin G, a glycoprotein that can be found in human plasma. According to the paper, this glycoprotein can induce the activation of antibody-dependent cellular cytotoxicity, that plays a major role in producing anticancer antibodies. 

A different proteomic biomarker, carbohydrate antigen 19-9 (CA19-9) is also offers useful information regarding the recovery from CRC. In an experiment, this biomarker was measured in patients before surgery and afterwards. The study concluded that after successful surgery, patients who developed recurrently colorectal cancer showed a significantly higher level of CA19-9 than the healthy ones [20].

Promising CRC screening results are presented in [21]. The work presents a successful method for colorectal cancer detection using a set of proteomic data: IQGAP3, which is the third member of the IQ-motif-containing GTPase-activating protein family, B7-H4 and COX-2. Although the efficiency of each biomarker varied depending of the tumor stage (T, N, M), the paper concluded that all three biomarkers proved to be reliable in tumor discovery. 

The notion of a computer aided diagnosis system (CAD) was first used in the end of the 20th century, by experts who relied on a computer to inspect medical images. These first rule-based systems used in the 1970s offered low-level image processing by using merely filters [3]. 

Today’s CAD systems often rely on artificial intelligence and deep learning, due to their high accuracy. Deep learning provides promising results not only on medical image analysis, but also on speech recognition, object detection, or language distinction and interpreting. From a medical point of view, there are two cases when experts should trust a CAD System as a second reader: in data and image analysis. Data analysis is usually performed with traditional machine learning algorithms, whereas deep learning is mostly used in image processing [3]. 

Taking into consideration the encouraging results of a wide range of studies on the role of biomarkers in CRC detection, in this research a computer aided diagnosis system was designed that detects colorectal cancer, based on an innovative dataset composing of both numeric and qualitative clinical data. The aim of the present study is to develop an intelligent computer aided diagnosis system that detects colorectal cancer using machine learning techniques. The novelty lies in the dataset composing of a unique combination of blood and urine analysis and qualitative data.

The paper is structured in seven chapters, in the following way: after the introduction, in Chapter two, the proposed methods used to design the intelligent CAD diagnosis system are presented; Chapter 3 exposes the two different types of experiments conducted to develop the system and the structure of the dataset containing 200 patients’ laboratory results augmented to 900 by classical methods; Chapter 4 discusses in details the two different approaches used to develop the software: classification problem with traditional machine learning and regression problem with neural networks; Chapter 5 presents the results obtained with the two methods: Chapter 6 reveals the preliminary test results of the best performing model; Chapter 7 draws the conclusions of the research and future development goals.

## 2. Proposed Methods

### 2.1. Machine Learning

Developing computer aided diagnosis systems is one of the many fields where machine learning algorithms gain popularity. Being a branch of artificial intelligence, machine learning refers to systems capable of educating themselves using data along with their previous experience, without taking direct instructions [22]. There are two main categories of machine learning algorithms: supervised learning and unsupervised learning. In supervised learning, labeled data (inputs and the corresponding outputs) is used to train the computer and create a model that later would be capable of correctly classifying unknown data. The most common supervised learning techniques used in medical research are linear and logistic regression [23]. Unsupervised learning suggests training a model on unlabeled dataset. Clustering is the best-known unsupervised learning technique, which implies the machine finding hidden patterns in a dataset and creating clusters based on these shared characteristics [7,24,25,26,27]. The use of unsupervised machine learning in medical applications is an emerging field. In [28], a computer aided diagnosis system is presented, that performs the correlation analysis between brain structure modifications, the clustering coefficient and the inspection of the Mini Mental Score, with an accuracy of 95.65%. In this research, supervised learning techniques were used to train a model on automatic colonic polyp detection system. 

Several studies have been conducted on using artificial intelligence in computer-aided cancer detection software aiming to reduce the risk of human error. One of the most common traditional machine-learning algorithms used in medical applications for data analysis are decision trees (DT). Being one of the oldest and most outstanding machine learning methods, decision trees have an architecture that can be easily understood and provides adequate results. Another machine learning technique gaining popularity in cancer detection software recently are support vector machines (SVMs). According to a study, SVMs were used in detecting breast cancer (with an accuracy of 95%, multiple myeloma with an accuracy of 71% and oral cancer with 75% accuracy) [29].

The most common technique used in medical image processing CAD Systems are convolutional neural networks (CNNs). A comprehensive study [22] compares the accuracy of deep learning models used in the detection of colorectal cancer in colonoscopy images. The models compared had a highly varying accuracy: 96.4%, 87.3%, 96% and 98%. These results prove the high efficiency of deep learning used in CAD diagnosis [22].

Several studies have been conducted on using deep learning for colorectal cancer detection in colonoscopy images. In [30], a novel dataset is presented that contains 3433 colonoscopy frames, divided into two categories: white-light and narrow-band images. Based on different deep learning approaches there are four different models constructed, trained and tested in the research and their performance on the PICCOLO and other two public datasets compared. The models are either based on backbones or encoder-decoder architectures. The study concludes that the four deep learning models have the best performance in colorectal tumor recognition on the novel PICCOLO dataset [30].

#### Validation Techniques

There are two validation techniques used for evaluating the performance of a machine learning algorithm: k-fold cross validation and holdout validation. 

K-fold cross validation consists of dividing randomly the dataset into k number of groups and training the model k times on the same dataset: in the k^th^ iteration the k^th^ group of data is used for validating the model and the other groups for training it. In this way, the ability of the model to make predictions on new data becomes measurable and the problem of overfitting is solved, since machine learning models using cross-validation are constructed only on a part of the dataset [31]. 

Holdout validation is based on partitioning the dataset into two subsets of different size. It is used for relatively large datasets. Training the model is performed on the larger subset, that usually consists of 75–85% of the entire dataset, while for validating the model the remaining 25–15% of the data is used [31]. 

### 2.2. Neural Networks

Neural networks are a subtype of machine learning, designed to model the human brain in order to solve complex classification problems, detect patterns in voice or images or detect cancer. They are composed of several layers, each of which contains a varying number of neurons. The first one, or leftmost is called input layer, while the last one is the output layer. The layers between the input and output are called the hidden layers of the network. If there is only one hidden layer then the network is referenced as shallow neural network. If there are more than one hidden layer, then the network is called deep neural network [32]. 

In the literature of CAD software numerous research have been carried out on artificial neural networks (ANNs) and deep learning. ANNs used to detect lung and breast cancer were presented in [22] as well as diagnosing pancreatic, colorectal and ovarian cancer according to [3].

#### 2.2.1. Feedforward Neural Networks

Regarding the direction of information flow, there are different types of neural networks. When the output value is obtained from the input and the following intermediate computations (the information flux is forward), the network is called feedforward. On the other hand, if there are feedback loops in the network where information from the output is fed back and the current output depends on previous outputs, the neural network is considered recurrent. In recurrent networks, the activation of some neurons can lead to the activation of a different set of neurons, causing a cascade stimulations. These types of neural networks have been less popular than feedforward networks due to lower level of efficiency of their learning algorithms. However, recurrent networks can emerge in the future and gain more attention than feedforward networks because they are better for modelling how human brain works [32].

#### 2.2.2. Training of a Neural Network

The training of a neural network refers to the adjustment of the weight and bias values until the desired, optimal performance is reached. This is achieved through solving the optimization problem: minimizing the cost function. In regression problems, usually this cost function is the mean squared error, minimized using gradient descent method. There are several optimization algorithms for neural networks, such as Levenberg–Marquardt, scaled conjugate gradient, bayesian regularization, gradient descent with momentum [25]. A recent study compared two of these training techniques: Levenberg–Marquardt with scaled conjugate gradient in order to obtain a multilayer perceptron neural network for the diagnosis of breast cancer. The research concluded that similar results were obtained with the two optimization algorithms regarding the speed and the accuracy, but the Levenberg–Marquardt algorithm had slightly better performance measures [29].

Additionally, the Levenberg-Marquardt is considered to be the fastest optimization algorithm for feedforward networks according to [31]. Another alternative for time-efficient training is the quasi-Newton algorithm, but the Levenberg–Marquardt optimization method is more reliable and ensures better performance on nonlinear regression (also called as function fitting) compared to pattern recognition tasks. In the Levenberg–Marquardt optimization algorithm, the gradient is a scalar obtained from the multiplication of a matrix and a vector. The first one is containing the partial derivatives of the cost function with respect to its parameters (weights and biases), while the second one is the vector of network errors. During the training of the neural network, the gradient calculated with the final parameters of the network should be minimized [31]. 

Concerning the architecture of neural networks there are several rules to consider to prevent overfitting. Overfitting is a common problem that occurs during the training of neural networks. It means that the model learns the noise from the training data, besides the useful information. In this way, the ability of the network generalization decreases. When constructing neural networks, a network should be large enough to build complex functions and achieve good performance, but small enough to prevent overfitting. In order to prevent it, the network should have considerably less parameters than the amount of data samples in the training set [31]. Moreover, according to [33], another way to prevent overfitting is increasing the size of the training dataset to the point where a considerably more generalized model can be built. Although being an overwhelming and time-consuming task, collecting a large training dataset can help to avoid underfitting as well, a phenomenon which can be provoked by reducing the complexity of the network to the point where it cannot make accurate predictions even during training [34]. 

### 2.3. Performance Measurement

An important point to consider when working with CAD (computer aided diagnosis) in tumor recognition (image processing/classification) is how high the level of confidence of a certain CAD method is. When diagnosing and classifying (benign/malignant) colonic tumor, radiologists would only trust CAD systems as a second reader, if their confidence level would tend to 0.9. A study [35] on confidence analysis compares 11 machine learning algorithms based on their accuracy, AUC (area under the curve) and probability outputs (P) regarding the recognition of a tumor and the nature of it (true positive/true negative), with the aim of determining their level of confidence. According to the results, the Bayesian Network (B-Net) and the Naive Bayes (NB) are the two most reliable machine learning algorithms from this point of view [35]. 

In contrast to this result, another study [36] suggests that ADTree (alternating decision tree) algorithm provides a machine learning model with the highest level of confidence. Additionally, a reliable colonic polyp detection system should ensure high sensitivity and specificity. Sensitivity measures the ratio between the true positives (the cases when the patient has tumor and the system detects it, denoted with TP) and the total number of patients having cancer. Specificity expresses the percentage of detecting true negatives (patients not having cancer, denoted with TN). In such way, a specificity of 0.9 implies that TNs are detected correctly in 9 cases out of 10 [7,28]. 

## 3. Experiments

### 3.1. The Data

**Figure 1:**
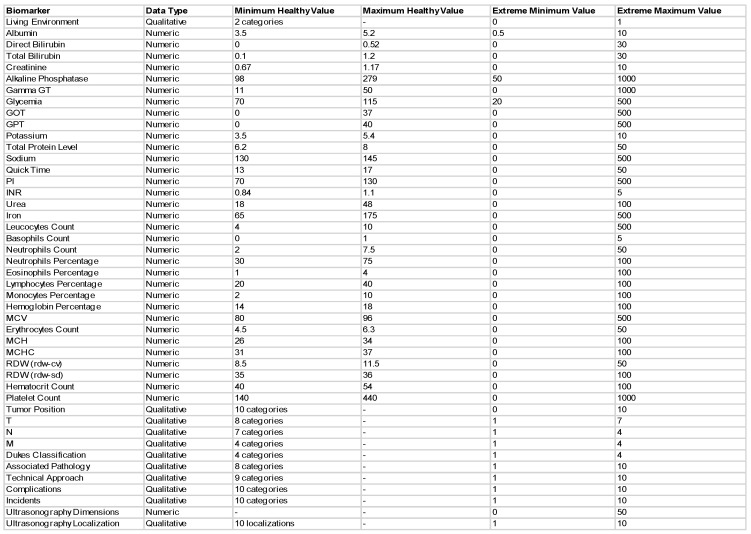
The structure of the novel dataset composing of numeric and qualitative clinical data.

In this research an innovative dataset was used with the aim of maximizing the efficiency of the colorectal cancer detection system, similar to the work presented in [37]. This novel dataset composed of the following 33 blood and urine data for each patient: albumin, direct and total bilirubin, creatinine, alkaline phosphatase, gamma GT, glycemia, GOT, GPT, potassium, total protein level, sodium, quick time, PI, INR, urea, iron, leukocytes, basophils, neutrophils count, neutrophils percentage, eosinophils percentage, lymphocytes percentage, monocytes percentage, MCV, hemoglobin percentage, erythrocytes count, MCH, MCHC, RDW (rdw-cv), RDW (rdw-sd), hematocrit and platelet count. Additionally, since professionals believe that sedentary lifestyle and western diet is mainly responsible for developing colorectal cancer [2], in this study, the environment in which the patients lived was taken into consideration when obtaining the diagnosis. Among these, 12 qualitative data types are used: tumor position, T, N, M, Dukes classification, associated pathology, technical approach, complications, incidents, ultrasonography-dimensions as well as localization. The structure of the dataset is shown in Figure 1. The dataset containing the 200 patients’ results and augmented to 900 by classical augmentation methods, was a table in Excel, which was then loaded into MATLAB® (MATLAB, 2020, The MathWorks Inc.: Natick, MS, USA) for further processing. A patient was represented by a row in the table, whereas variables were indicated by columns. Additionally, there were added to the table the minimum and maximum values of the healthy range in the first two rows of each column. Therefore, the resulting dataset had 902 rows and 45 columns. 

### 3.2. CAD Systems Designed

The development of the computer aided diagnosis software was performed with the help of the machine learning and neural network toolboxes of the MATLAB®. For training a model with any of them, two types of input variables were needed: a set of predictors and one response variable. 

In the first phase of the research, machine learning models were trained using the Classification Learner application of the Machine Learning toolbox to solve a binary classification problem: from continuous and qualitative input data, obtaining a binary (true/false or healthy/suspected to have cancer) diagnosis. In total, six types of models were trained using two different validation techniques: k-fold cross validation and holdout validation. Using k-fold cross validation three different approaches for selecting k were implemented: one for a relative small k (taking the value 5), one for a medium k (k = 25) and one for a large number of folds (k = 50). Similarly, three different methods were tested regarding the percentage of data held out (5%, 15%, 25%) in holdout validation. At the end of all 6 experiments, the best performing models resulting from each were compared.

In the second phase of the research, shallow and deep neural networks were trained using the Neural Network toolbox to solve the regression problem, which provided experts with a continuous response representing the probability for the presence of colorectal cancer.

In addition to the novelty of the dataset, the most outstanding difference between this research and the state of the art lies in the discussion of the problem: there are two thoroughly different approaches discussed. Not only the nature of the two problems differ (classification and regression problems), but also the methods used and the models constructed.

## 4. Discussion

### 4.1. Classification Problem Solved with Traditional Machine Learning

#### 4.1.1. Data Preprocessing and Labeling

Preprocessing the data was performed in terms of a data labeling process. It consisted of categorizing each record from the dataset as unhealthy (denoted by 1) or healthy (0). Since there were given the intervals of healthy values for each variable, values outside the healthy interval were labeled as 1 (the true value indicating the possibility of having a tumor) and values from the normal ranges had 0 as label. Taking into consideration that people living in urban environment are being associated with higher risk of cancer, in the case of the variable representing the rural or urban provenance of the patient, the urban origin was denoted with label 1 and the rural otherwise. Similarly, since there were 10 different categories describing the number of associated diseases of a patient, the following concept was applied: if the person had no other pathologies that could possibly influence the growth of colorectal tumor, then he was considered “healthy” from this point of view. Therefore, the first category denoted with 1 was labeled with 0 and all the other categories indicating the presence of other diseases or risk factors as 1. The other qualitative variables were labeled in a similar manner. After the labeling process, a label-matrix of Boolean values was obtained having 900 rows and 45 columns. 

#### 4.1.2. Obtaining the Response Variable

##### Absolute Deviation

Attaining the response variable was accomplished in two steps. On one hand, the first step was finding (for every data record) the value of a variable measuring the probability that a patient had cancer. The workflow was the following: for each data all 45 variables were examined one after the other. For each variable there was calculated the absolute deviation and consequently the extent of this deviation showing how far is the value of a variable outside the healthy range was comparative to the healthy value, expressed in percentage.

For a 100% healthy patient with all the variables within the healthy range (and therefore, all the labels having the value 0), the probability for tumor was 0. However, if there were predictors suggesting that the person was unhealthy, the probability was calculated in a more complex way. Two factors were then considered: how significant the deviation from the healthy range was and how influential or decisive the predictor was in determining whether the patient had tumor. According to this, the calculation of the first factor was performed using the formula of the absolute deviation and then converting this value into a percentage based on the magnitude of it. These values were stored in a column vector and each row of this column vector was corresponding to a different patient. In the case when the data record had a variable within the healthy range, both the absolute deviation and consequently, this percentage had the value 0.

##### Weight of Each Predictor

The second factor in computing the probability of a patient having cancer was the influence each variable had in this decision. In order to distinguish between predictors with more and less importance, each predictor was assigned a weight. For instance, if the elevated level of glucose in the blood represented a higher predisposition to cancer than the elevated level of potassium, the latter had a smaller weight associated with. Therefore, after finding the percent of deviation in the previous step, there was calculated a weighted average with the help of these “weights of risk”. Finally, another percentage was obtained for each data, telling in a more precise way the actual level of risk or probability of having colorectal tumor.

Taking into consideration that in the research a completely novel dataset was used with a unique combination of variables, there were no previously defined principles regarding the degree of contribution each individual variable had to the final diagnosis. Hence, several calculations were performed to establish the weights.

In the first approach, determining the weights was based on the presumption that each variable had approximately the same impact on the diagnosis. The weights took values in the range of 0.015 and 0.05, but the majority revolved around the value 0.029. This value was chosen with the purpose of obtaining approximately 1 as the sum of weights.

The second approach of finding the appropriate weights was based on the expansion of the (healthy and unhealthy) ranges of each variable. Firstly, the relative difference (or percentage of change) was calculated for both the lower and upper extremities of the intervals. This quantity measured the absolute difference between the healthy (or reference value) and unhealthy limits, divided by the reference value (the boundary of the healthy range). Since two relative differences were obtained for each variable: one for the minimum and one for the maximum values, the arithmetic mean of these differences was calculated. Based on this principle, there were obtained weights proportional with the possible deflection of the variable. 

**Figure 2:**
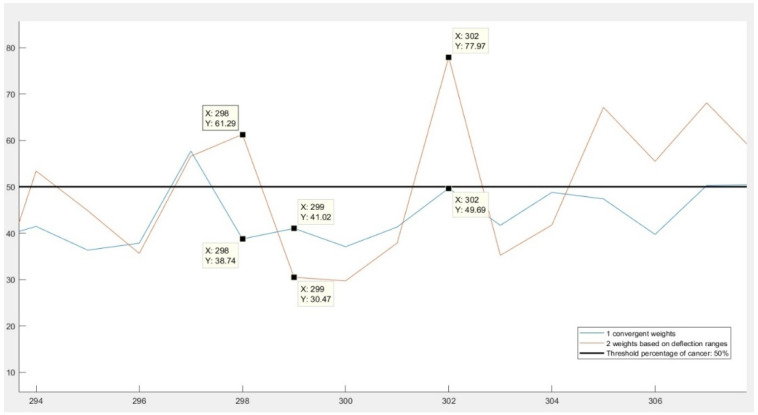
Comparison of the results obtained using convergent weights and weights based on deflection ranges.

Although this second approach seemed more reasonable than the first one in theory, the results were not confirming these assumptions. In Figure 2, a comparison of the results can be seen. The differences are exemplified with three records. Both the 298th and 299th data had approximately the same results: they had the same number of variables in the healthy range and some variables outside of it. Even the amplitude of deviations from the healthy ranges were similar, except for one fact: their variables with “unhealthy” values were different. It can be observed on the figure that using convergent weights, both patients had about the same results: 38.74% and 41.02% of cancer. However, according to the results obtained from the second approach, the first patient was well above the threshold value 50%, therefore considered unhealthy, while the second one was labeled as healthy. Additionally, not only the diagnosis was different for them, the two records also had excessively diverse probability of colorectal cancer: 61.29% and 38.74%. This behavior was due to the huge differences in the order of magnitude of the weights: some variables had considerably greater impact on the final diagnosis than others.

However, in the case of data number 302, the second approach proved to be more appropriate. This patient had values exceeding the accepted values by 4–5 hundred percent in several variables. The second method interpreted the results as a 77.97% of probability of cancer and labeled the person as positive, while the method with convergent weights labeled him as healthy, since the probability (49.69%) in this case did not exceed the threshold of 50%. 

In conclusion, both approaches provided reliable results only in some cases. The convergent weights did not emphasize sufficiently the differences between healthy and potentially unhealthy patients and in many cases indicated an opposite diagnosis than working with the proportional weights. The weights based on deflection ranges were dependent on a few particular variables in an unbalanced way.

Misdiagnosing a healthy person as “at risk of having colorectal cancer” is called false positive, while labeling a patient with high risk of cancer as healthy is false negative diagnosis. Taking into consideration that fact that a computer aided diagnosis system should, in the first place, reduce the risk of cancer miss-rate and draw the attention of the doctor to any anomaly, the number of false negatives should be reduced to the maximum extent. Therefore, to avoid the huge differences in the order of magnitudes of the weights and reduce the number of false negatives, a third approach was imposed from the combination of the previous strategies. These “final” weights were numbers between 0.01 and 0.03, so convergent, but also expressing in their values the importance of each variable.

**Figure 3:**
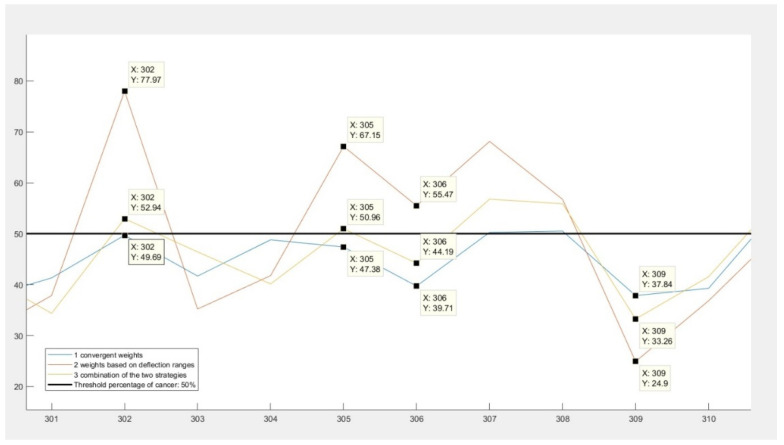
Third approach: combining the two previous strategies.

In Figure 3 is shown the results given by these weights. There are two important behaviors to be observed. Firstly, that the final results represented by the yellow line are between the results given by the other two strategies, regardless of the nature of the diagnosis or the sign of difference between them. The second behavior of the final strategy can be examined in the case of data number 305 and 302. These records had results indicating that they had a chance of colorectal cancer, but the convergent weights (represented by the blue color in the figure) gave false negative results.

##### Normalizing the Continuous Response Variable

An important aspect to be considered when determining the probability of cancer was the maximum value of this variable. Since the probability is always expressed in percentage, the chance of a person having colorectal cancer should vary between 0 and 100%. Taking into account that in the case of almost every variable the possible deflection from the accepted healthy range exceeded 100%, the final percentage measuring the risk of cancer could also exceed 100%. This problem was solved in two steps. 

On one hand, the maximum possible value was calculated that this percentage could take. Then, a fictive patient was generated who was having 100% cancer: all of his results were taking the maximum possible values. Afterwards, the probability of cancer was computed for this patient. 

**Figure 4:**
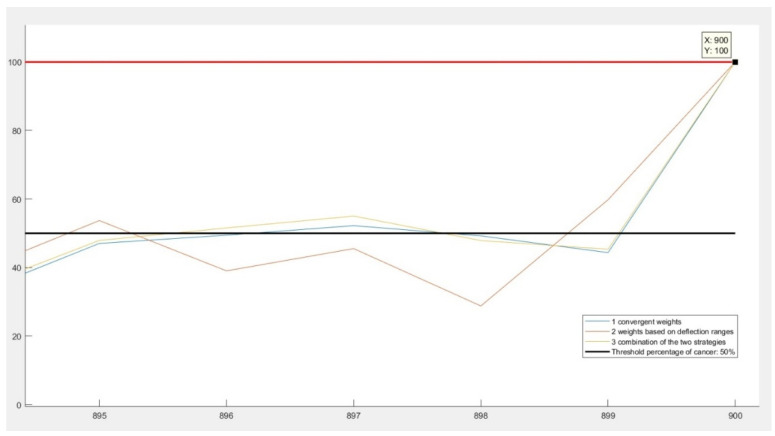
Normalization of the resulting percentages.

On the other hand, once created and inspected the worst-case scenario, all results were normalized with respect to this percentage. Normalization in this case meant dividing each result with this probability of worst case. In Figure 4, it is illustrated how, in the case of the last (fictive) patient, all three algorithms converge to 100%, despite of giving different results for previous patients.

It is important to mention that in the present, this colorectal cancer diagnosis software has not been tested profoundly by medical experts yet. Thus, the posterior adjustment of the weights might be necessary based on medical knowledge and experience. Moreover, by modifying these values of the weights, the machine learning model and the final diagnosis are modified as well. 

#### 4.1.3. Labeling the Response Variable

As previously mentioned, the first goal of the research was solving the binary classification problem. Therefore, for the training of the model a labeled response variable was needed, representing the answer of the model to the input data. A person was considered unhealthy and suspected to have colonic cancer if the variable expressing the probability of colorectal tumor had a value above 50%. In this case, the response variable would take the value 1 and if the value was below 50%, the response was 0. This boundary value was determined based on a discussion with the expert.

#### 4.1.4. Developing the Machine Learning Model 

After obtaining the predictors and the response variable, several models were trained using the Classification Learner toolbox in MATLAB®. This toolbox offers the possibility of training models using k-fold cross validation and holdout validation as well. Therefore, models were trained using two different validation techniques, three different approaches for each. At the end of all six experiments, the best performing models resulting from each were compared.

#### 4.1.5. K-Fold Cross Validation

The first validation technique used was k-fold cross validation. In this research three different approaches were tested: 5-, 25- and 50-fold cross validation. The accuracy and the efficiency were both considered when comparing the resulting models, with the intention of finding the best performing one.

##### 5-Fold Cross Validation

**Figure 5:**
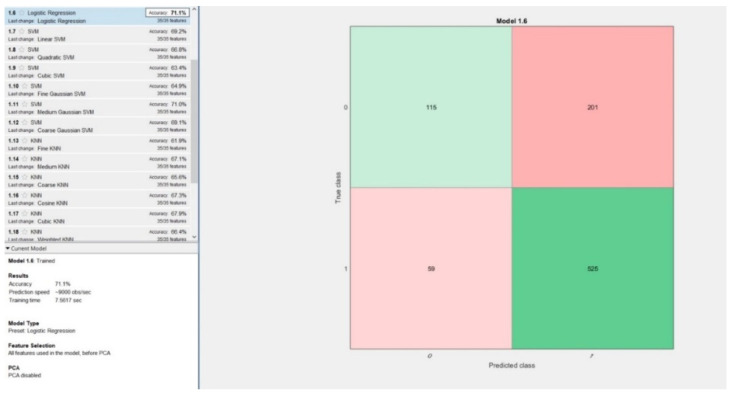
Best performing model obtained using 5-fold cross validation: Logistic Regression.

The first approach was separating the dataset in 5 folds, k taking the value 5. The best performing model based on this approach was logistic regression, having an accuracy equal with 71.1% and training time 7.56 s. Inspecting the confusion matrix resulting from this model in Figure 5, it is observable, that even though the number of false negatives (59) is low relative to the number of false positives (201), it is not an outstanding performance. The percentage of false negatives in this model was equal to 6.55%, which is unacceptably high in computer aided cancer diagnosis systems.

##### 25-Fold Cross-Validation

The best performing model using 25-fold cross validation was Logistic Regression with an accuracy of 71.8% and a training time of 9 s. The other resulting models trained with this validation technique had an average accuracy of 67%. It is important to consider that the number of false negatives was still relatively high, 56, only slightly smaller than in the previous experiment.

##### 50-Fold Cross Validation

For k equal to 50, using 50-fold cross validation, two models were obtaining the same, best accuracy of 71.3%. These models were: logistic regression and medium Gaussian support vector machines (SVM). Though the accuracies were equal, the training time for Logistic Regression model was half (16.5 s) of the training time of the SVM (30 s) model. Taking into consideration the number of false negatives and positives, the logistic regression model had a higher number of false negatives (56) and lower number of false positives (202) than the SVM model (36 false negatives and 222 false positives). In cancer diagnosis, false negatives are more dangerous than false positives; therefore, the SVM model was considered more reliable.

#### 4.1.6. Holdout Validation

Regarding the percentage of data held out three different methods were tested in this study. The resulting accuracies of the models, training time and number of false negatives were compared at the end.

##### 5% of Data Held out for Validation

**Figure 6:**
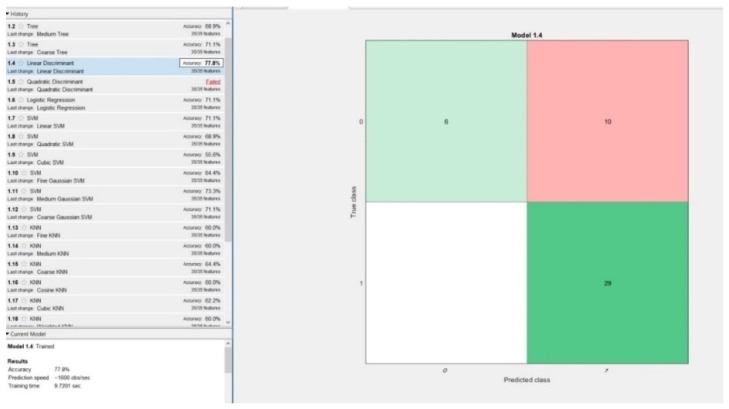
Best performing model obtained using 5% hold out validation: linear discriminant.

In the first phase, the models were trained on 95% of the data, while only 5% of it was left out for validation. The best performing model was linear discriminant with an accuracy of 77.8% and a training time of 9.7 s. Surprisingly, there were no false negatives during the model validation process, which was performed using 45 random data records, 5% of the 900 samples, Figure 6. 

##### 15% of Data Held out for Validation

The most common proportion of data held out when using holdout validation is 15%. Applying this rule, the following results were obtained: the highest accuracy equal to 70.4% was achieved by medium Gaussian support vector machines (SVM) model. The training time was 2.44s and the number of false negatives were 5 out of 135 test samples.

##### 25% of Data Held out for Validation

In the third phase, the models were trained on 75% of the data and the remaining 25% was left out for validation. There were three different models attaining the same accuracy (70.2%), but regarding the other two performance measures (training time and number of false negatives), they had different results. The best performing model was the medium Gaussian SVM, with a training time of 1.53 s and six false negatives, only 2.66% of the training dataset that contained 225 samples. The other two models, logistic regression and bagged trees had 10 and 12 false negatives and higher training times as well. 

### 4.2. Regression Problem Solved with Artificial Neural Networks

#### 4.2.1. Reasoning of the Second Approach

A cancer detection software should have an accuracy above 89% (between 89% and 95%, more precisely) in order to provide the experts a reliable and useful second-opinion [32]. After obtaining the results with the classification learner toolbox we concluded that even the best performing model (linear discriminant model obtained from using 5% holdout validation) had an accuracy equal to 77.8%. This was a relatively low level; therefore, the model was not acceptable to be used in a computer aided cancer diagnosis software, suggesting that solving only a classification problem might not provide with adequate results. This problem made the finding of a different approach necessary. 

Firstly, for the improvement of the CAD diagnosis system, there was made a change in nature of the input data. In the first part of the research the labeled dataset was used as input for the construction of the machine learning models. Therefore, not only the output was a binary variable, but the inputs as well. In the second part of the research the input data used for constructing and training the neural networks was continuous to prevent information loss during conversion from continuous to discrete variables. The percentage of deviation from the healthy range of each input variable was taken into consideration in the construction of the response variable.

On one hand, one cannot tell if a person who is 49.9% sure to be having cancer is healthy and if this percentage is 50.1%, then he is unhealthy. Such diagnosis method could lead to a considerably large number of false negatives, so in order to obtain a less exact, but more realistic diagnosis, the classification problem was transformed into a regression problem, since medical science is not an exact one based on zeros and ones. Later, this value of the probability of cancer in percentage was displayed on the user interface. In the regression problem, a linear response was obtained, representing the probability of the existence of cancer. The labeling of the input variables using Boolean values was necessary in order to obtain the continuous response, but these binary predictors were not used as inputs during the model construction and training, as in the first part of the research.

On the other hand, taking into consideration the fact that deep neural networks perform better on large datasets than basic machine learning algorithms, solving a regression problem was not performed using the Regression Learner, but rather with the Neural Network toolbox in MATLAB®.

#### 4.2.2. The Architecture of the Network

According to [38], the performance of a deep learning algorithm depends on the architecture and the parameters of the neural network. Therefore, in order to obtain the highest possible accuracy for the CAD system, 10 different network architectures were inspected and their performance results compared. A similar approach is presented in [33], where several deep and shallow neural network models are compared with the aim of enhancing the sensitivity, specificity and accuracy of the CAD system. The study compares three different CNN models and trains them for 30 epochs.

In this study, there were 10 networks trained with different architectures and parameters and their results compared. The type of networks was feedforward with backpropagation in all experiments, but the number of layers and neurons were varying. Both the number of layers and the number of neurons in the first nine experiments were taking three types of values: low, medium and large. The last network was constructed using the conclusions drawn from the performance of the previous nine networks. The performance function was Mean Squared Error (MSE) and the Levenberg–Marquardt was chosen as the training algorithm. 

Along with the performance function (MSE), four other performance parameters were examined when evaluating the performance of the networks. These parameters are: number of iterations on the whole dataset during training (called number of epochs), training time measured in seconds, the gradient and the accuracy. Although the main goal of the training was minimizing the performance function (MSE) and obtaining the highest possible accuracy, the minimization other two performance measures was a secondary objective. The division of data between training, validation and test set was the following: 60% used for training and 20% for both network testing and validation. The number of validation checks was set to six during the training of all the 10 networks. This boundary value is offering protection against overfitting, since it sets the limit of how many iterations can be performed on the training dataset while obtaining the same value of the performance function [19].

The networks were trained on a computer with i7 9700K, 4.7 GHz Turbo Boost CPU and a ddr4 memory of 16 GB. It is important to mention that the resulting performance measures of the networks presented below may vary depending on the hardware on which the training is performed.

#### 4.2.3. Examination of the Networks

The first three types of neural networks were shallow neural networks having only one hidden layer and a varying number of neurons on that hidden layer. 

**Figure 7:**
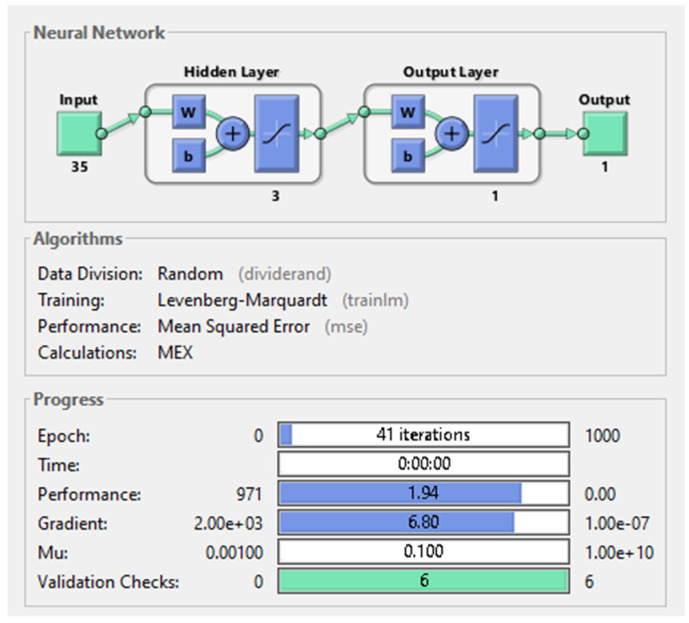
Shallow neural network with 1 hidden-layer and 3 neurons on it.

In the first case, when the hidden layer had only three neurons, an MSE equal to 1.94 was obtained after training the network. Despite that the total number of iterations on the dataset was 41, the training time lasted 0 s and the gradient was equal to 6.8. The network architecture and parameters can be seen in Figure 7 below.

**Figure 8:**
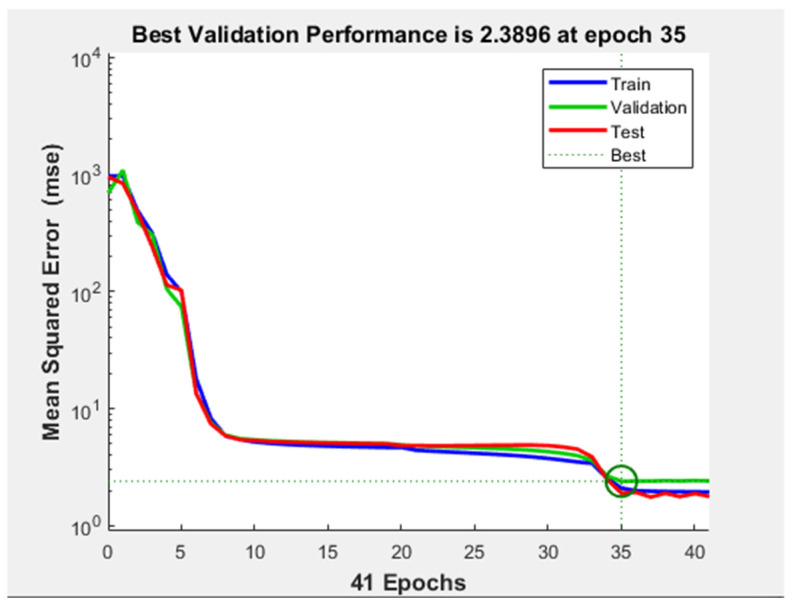
Performance plot the shallow neural network with 1 hidden layer and 3 neurons on it.

Regarding the evaluation of the MSE with respect to the number of iterations, in Figure 8, it is illustrated that the minimum of the cost function was almost reached after epoch number eight. However, the training did not stop because the validation performance was not constant for six iterations (validation checks). After the 24th epoch, the test curve started to increase with respect to the validation curve, but this phenomenon cannot be considered overfitting, since the difference between the two curves was not significant.

**Figure 9:**
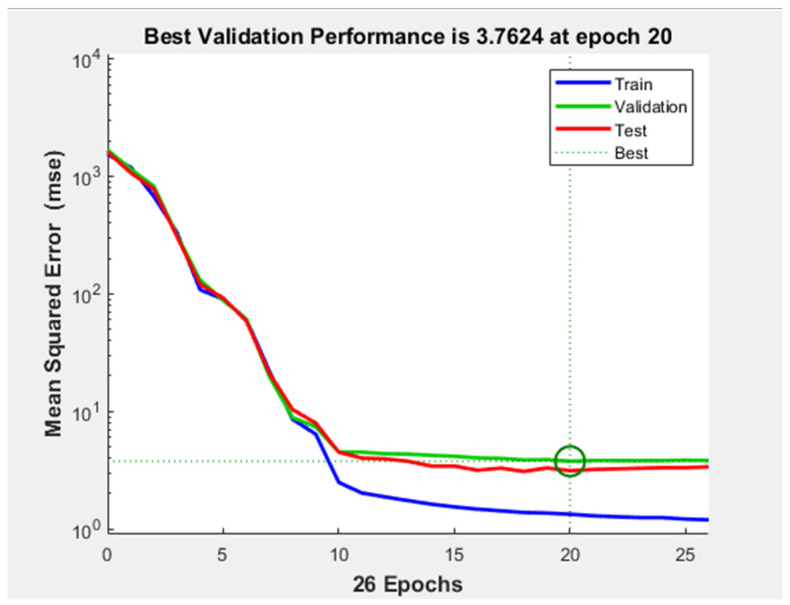
Performance plot the shallow neural network with 1 hidden layer and 10 neurons on it.

In the second case, a network with 10 neurons on the one hidden layer was constructed. The MSE of this network is similar to the one obtained with the previous network, as well as the training time which lasted 0 s. However, the training stopped after half as many epochs (26) than in the previous case and the gradient was taking a larger value, namely 10.7. In Figure 9, the performance plot of the network can be seen. The test and validation curves are similar, but in the test curve a smaller MSE is obtained. This indicates that no overfitting occurred during training. The validation curve reached the minimum of the MSE at epoch number 20.

In the third case, a shallow neural network having 20 neurons was constructed. This network presented the highest MSE among all three shallow neural networks: 3.49. The number of epochs was 32, but the best performance was reached after 26 iterations. The only significantly better performance measure obtained with this shallow neural network was the gradient which had a value of 2.62.

Taking into consideration the results obtained from training these three shallow neural networks one can see that the MSE was not minimized excessively, remaining in the 0–10 interval.

The next three networks were built on a 5-hidden layer architecture and therefore were called deep neural networks. The number of neurons was chosen having the same values as in the case of the shallow neural networks.

Evaluating the results obtained after training the first network with five hidden layers (three neurons on each), was concluded that this network had the worst performances. First of all, the minimum of the MSE was 10 times higher than the ones obtained with any of the shallow neural networks. This was an unexpected result, taking into consideration the fact that in general, increasing the complexity of the network and the number of parameters (weights) of it should yield better results. Secondly, the gradient minimization (equal to 57.1) of this network was also the least successful among all 10 networks. Despite these inadequate results provided by the network, the performance plot indicated that no overfitting or underfitting has occurred.

The second deep neural network architecture having 10 neurons on each hidden layer yielded similar results as the shallow networks. The MSE was equal to 2.15, the gradient 6.07 and the training time lasted 0 s. The only improvement was observed examining the total number of iterations on the dataset. The value of this performance parameter was 16, significantly less than in shallow networks, however, not affecting the training time.

The best performing model among the first 9 ANNs was the deep neural network having 5 hidden layers and 20 neurons on each layer. Despite of the relatively low number of iterations on the dataset (15 epochs) the training time was 4 s: significantly higher than in the previous, more simple models. It can be noticed that the training time was closely related to the complexity of the network: the more neurons and layers it had, the more the training time lasted. The gradient obtained with this network was 6.45, that, comparing to the results of other networks had an average value. Regarding the most important performance measure, the mean squared error (MSE), this neural network had the second-best results in minimizing it. The value achieved: 0.000623 was 10 times smaller than the MSE obtained with the second-best performing network (having 10 layers and 20 neurons on each layer) and 50,000 times smaller than the MSE obtained with the worst performing neural network (having five layers and three neurons). 

The next three deep neural networks were built on the principle of having 10 hidden layers and a varying number of neurons on those layers. Their evaluation is presented below.

With three neurons on each of his layers, the neural network having 10 hidden layers yielded an MSE comparable to the mean squared errors obtained with shallow networks. The training time lasted 0 s, equivalent to the majority of the other networks. Regarding the other two performance measures; the number of epochs and the gradient, the network provided rather inadequate results. Among the first nine networks trained, in this one the gradient took the second largest value: 25.5. Moreover, the most iteration on the dataset was performed by this network with a number of epochs equal to 56. 

Inspecting the performance plot of the network, the following conclusion was drawn: the training was successful in terms of avoiding over and underfitting, but from the point of view of the minimization of the cost function it offered mediocre results.

Increasing the number of neurons on each hidden layers of the 10-layered network granted significantly better results. The mean squared error provided by it was in the interval of [0;1], taking the value 0.508. The number of iterations on the dataset was also the third lowest value: the training stopped only after 20 epochs. The other two performance parameters were above the average: the gradient was equal to 14.3, while the training time took 2 s.

**Figure 10:**
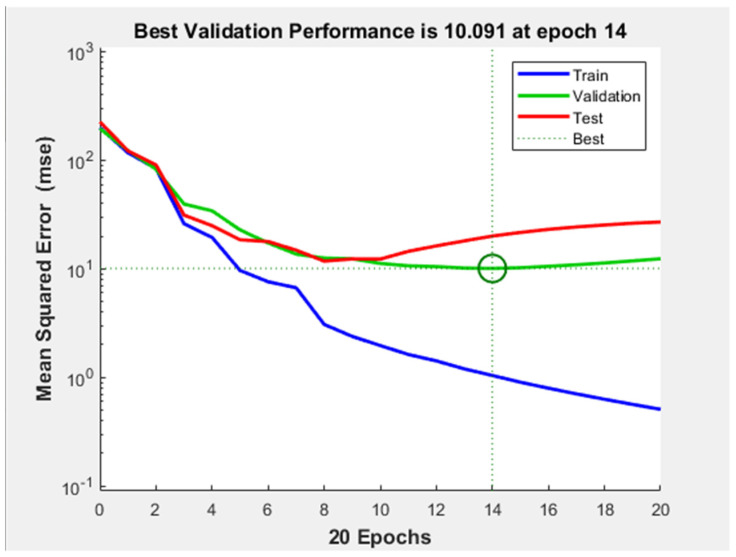
Performance plot of neural network with 10 hidden-layers and 10 neurons on each hidden-layer.

Despite of the acceptable performance of the network, the performance plot (Figure 10) indicates that a considerable overfitting occurred during training: after epoch number 10 the test curve starts to increase severely, while the validation curve continues to descend. This demonstrates that the model learned the noise from the training dataset.

**Figure 11:**
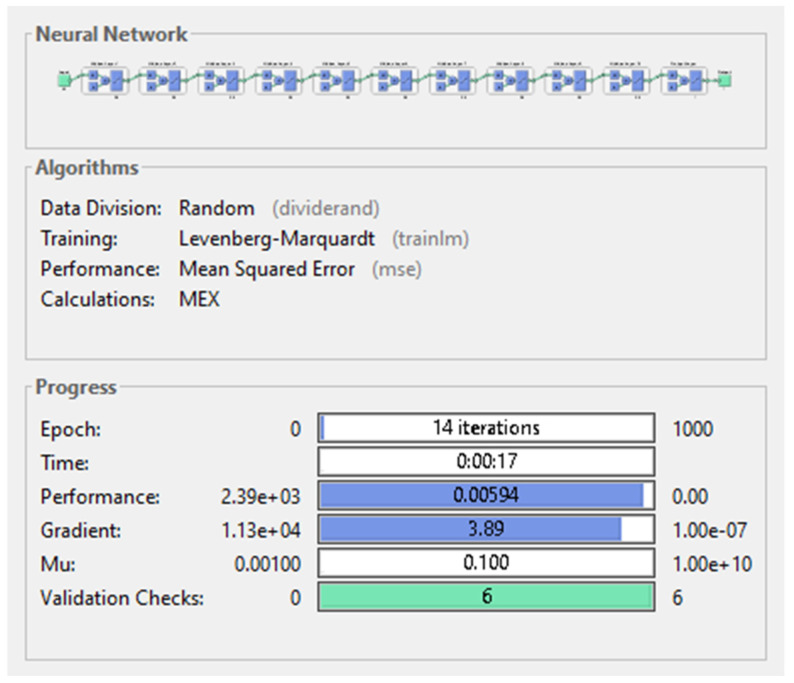
Neural network with 10 hidden-layers and 20 neurons on each hidden-layer.

The last among the first nine networks trained was the neural network having 20 neurons on each of its 10 hidden layers. This network had outstanding performance on almost every level: the MSE presented by it had the second lowest value (0.00594) which was a hundred times better than the MSE provided by the third best performing model, but still 10 times higher than the lowest MSE yielded by the best performing model. The gradient was taking the second lowest value (3.89), illustrated in Figure 11 below. Despite of requiring the lowest number of total passes on the data (14 epochs), the training of the network lasted 17 s, becoming the longest training duration measured on the first nine networks.

**Figure 12:**
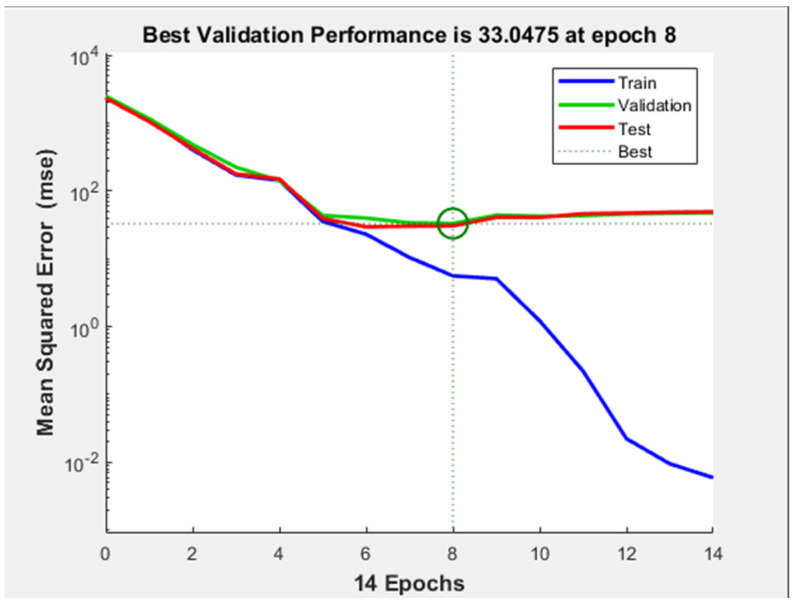
Performance plot of neural network with 10 hidden-layers and 20 neurons on each hidden-layer.

Examining the performance plot of the network (Figure 12), it can be observed that no over or underfitting has occurred during training and the minimum MSE was approximately reached only after the 4th epoch.

Based on the knowledge gained from training the first nine networks, a 10th deep neural network was designed in the attempt to maximize the performance. The optimized structure of it was constructed based on two aspects deducted from the experiments: for the minimization of the MSE the number of neurons must be higher than the number of hidden layers; for finding the minimum possible training time, the number of hidden layers has to be kept at a medium value. Based on these arguments, the 10th ANN had five hidden layers with 40 neurons on them. 

**Figure 13:**
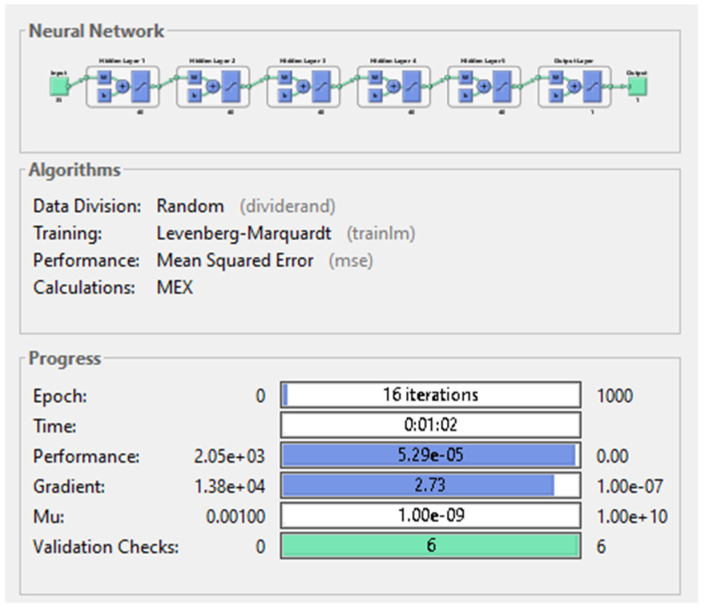
Deep neural network with 5 hidden-layers and 40 neurons on each layer.

The results obtained after training the last model were confirming the conclusions drawn from the previous experiments. Due to the high complexity of the network, the training time exceeded one minute, despite of the low number of epochs needed to complete the training. In minimizing the MSE, this network was more than 10 times more successful than the best performing ANN from the first nine experiments, reaching the value 5.29 × 10^−5^. Outstanding result was obtained by the ANN regarding the gradient as well: 2.73, the second smallest value among the networks. Figure 13 illustrates the structure of the best performing deep neural network.

## 5. Results

### 5.1. Classification Problem

After training the classification algorithms, the resulting models’ performances were compared. The performance measures inspected were the following: the accuracy of the models, the training time, the percentage of false negatives, the sensitivity, specificity and the precision. The accuracy, sensitivity, specificity and precision are performance measures that depend on the number of true/false positives and true/false negatives, which describe the ability of the system to predict correctly the outputs. The formulas of the four performance measures are the following [28,38]:

$$Accuracy=\frac{TP+TN}{TP+TN+FP+FN}$$

$$Sensitivity=\frac{TP}{TP+FN}$$

$$Specificity=\frac{TN}{TN+FP}$$

$$Precision=\frac{TP}{TP+FP}$$

Evaluating the results obtained using k-fold cross validation, one can notice that the model with the highest accuracy of 71.8%, highest specificity of 37.34% and highest precision of 72.72% was obtained with 25-fold cross validation. This model was Logistic Regression with a training time of 9 s, a considerably less duration than the training time of models with 71.3% accuracy, obtained from 50-fold cross validation. Regarding the number of false negatives, the best performing model was the medium Gaussian SVM with a percentage of 4% false negatives from the 900 records. This is 2.22% less than the percentage of false negatives in the case of the two logistic regression models (for 25- and 50-fold cross validation). 

Table 1, shows the results obtained from applying k-fold cross validation. The column with header “% of false negatives” shows the number of false negatives proportional to the number of samples in the validation dataset. In the case of k-fold cross validation, the percentage is calculated relative to the whole dataset containing 900 samples.

Comparing the results obtained from all 3 holdout validation techniques, one can see that the highest accuracy of 77.8%, sensitivity of 100% and specificity of 37.5% along with the lowest number (0, more precisely) of false negatives was obtained using 5% hold out validation, with the linear discriminant model. Additionally, studying all six resulting models (from both cross and holdout validation), lead to the conclusion that the linear discriminant model obtained from 5% holdout validation was the best performing model.

Regarding the average values of the performance measures, the following conclusions were drawn: using k-fold cross validation, the best resulting models had an average accuracy equal to 71.4%, average training time of 15.52 s and average percentage of false negatives from test dataset equal to 5.59%. The average sensitivity of the six models was 94.04%, the specificity 1.45% and the average precision equal to 71.71%.

Holdout validation techniques gave better results regarding the first four performance measures, Table 2. The average accuracy of best performing models was 72.8%, the average training time of models lasted 4.55 s and there was a 2.12% of false negatives. K-fold cross validation yielded better results regarding the specificity and the precision. In conclusion, the holdout validation technique proved to give slightly better results than K-fold cross validation.

Our results are comparable with similar results obtained in the domain. For example, in paper [23] are obtained accuracy between 66.9% and 74.51%, sensitivity between 49.36% and 66.82%, specificity between 80.42% and 80.97% and precision between 68.12% and 74.11%. In [22], a method proposed by Blanes-Vidal et al. yielded with better results: an accuracy above 96%, a sensitivity of 97% and a specificity of 93%.

Another work conducted in colorectal cancer detection in colonoscopy [38] compares three different models with the following results: the first model had an accuracy of 68.91%, sensitivity of 66.27% and specificity of 71.94%, the second model attained an accuracy of 66.50%, sensitivity of 87.43% and specificity of 42.47%, while the third model yielded an accuracy of 52.60%, with a sensitivity of 19.55% and specificity of 90.55% [38].

### 5.2. Regression Problem

After training all 10 neural networks the following deductions were made: enhancing the complexity of the networks by adding more layers or neurons did not necessarily lead to improved performance. In fact, if the number of neurons was smaller or equal to the number of hidden layers, led to poor performance, implying that the number of neurons should be increased along with the number of hidden layers. To exemplify this aspect, the performance measures of the shallow neural network having three neurons on its hidden layer can be compared with the performances of the deep neural network having five layers and three and 10 neurons on them (Table 3). 

Similarly, the minimization of the gradient was closely related to the number of neurons the ANN had: a network with more nodes on its hidden layers minimized the gradient more efficient.

In Table 3 an interesting relationship can be observed between the efficiency of the minimization of the cost function and the training time. In the four best performing networks (producing the smallest MSE) the training time was measurable in seconds: it varied between 2 and 62 s. However, in the case of the other six networks this duration (0 s) was not perceptible for the human eye. The detailed results regarding all the performance measures of the networks are accumulated in Table 3 below. 

Comparing the results obtained with the best performing model with the results presented in [39] we can conclude that the MSE obtained in this research is significantly lower than the MSE obtained in [39], which is equal to 0.01, reached after 2000 epochs. 

After concluding that the MSE obtained with the best performing deep neural network was equal to 5.29 × 10^−5^ an accuracy of 99.106% was obtained for this model. This result indicates that the CAD system designed met the expectations of a reliable intelligent cancer diagnosis system. 

Comparing this result with other diagnosis systems presented in the state of the art also indicates its efficiency: In [39] several models were compared in the literature of CAD diagnosis systems with the accuracies: 93.85%, 92.45%, 93.08%, 92.58% and 97%. In [35], the accuracies vary less, between 92.97% and 99.26%. Moreover, similar accuracies were obtained in work [38]. Therefore, the diagnosis system presented in this research represents a contribution to the evolution of computer aided diagnosis.

## 6. Testing the System

**Figure 14:**
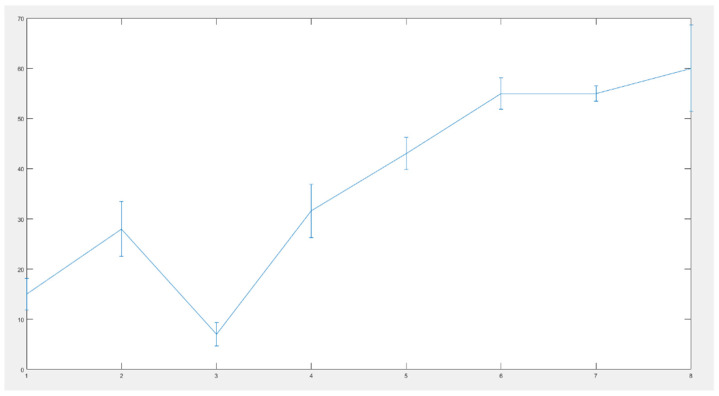
Difference between the two diagnoses: error bar graph.

The best performing deep neural network was tested in several scenarios: medical experts examined a reduced set of eight patients and estimated the probability of having tumor. After colonoscopy was taken, this percentage was reevaluated and adjusted, if needed. Then, the data of patients was loaded into the CAD system. The difference between the two diagnoses is indicated on Figure 14 below:

The vertical lines on Figure 14 indicate the deflection of the human diagnosis from the diagnosis specified by the intelligent system. All errors are under 10%, indicating a good behavior of the developed diagnosis system. 

## 7. Conclusions and Future Goals

Taking into consideration the results obtained from solving the binary classification problem with traditional machine learning algorithms, a cancer detection software should have a considerably higher accuracy than 77.8%; therefore, the machine learning model resulting from the classification problem cannot serve experts with a reliable second opinion. Moreover, regarding the nature of the output variable, a cancer detection software should provide experts a continuous response in terms of a percentage rather than a discrete variable in order to avoid misdiagnosis. Therefore, in the second approach the binary classification problem was turned into a regression problem.

In the attempt of finding a solution to the regression problem, a different machine learning technique was exploited: artificial neural networks. There were 10 networks trained with different architectures. The networks presented a varying performance regarding the minimization of the cost function, the mean squared error. The best performing deep neural network having five hidden layers and 40 neurons on each hidden layer provided significant results in the minimization process of the cost function, ensuring that the MSE had an order of magnitude of 10^−5^ and an accuracy equal to 99.106%.

In conclusion, deep neural networks are both more reliable and more efficient in colorectal cancer detection than traditional machine learning algorithms. The optimal neural network should have tens of neurons to ensure high performance and several hidden layers, while keeping the training time as low as possible.

The innovative structure of the dataset used in this study offers a new perspective for the non-invasive colorectal cancer diagnosis. The presented novel combination of the numeric data from blood and urine analysis with the qualitative data ensures a more detailed and precise diagnosis of the patient. The future goal is to enhance this intelligent colorectal cancer detection software with an image processing functionality in order to detect cancer in colonoscopy frames.

## Figures and Tables

**Table 1:** Results obtained using k-fold cross validation.

K-Fold Cross Validation	Performance Measures of Models						
	Best Performing Model	Accuracy	Training Time	% of False Negatives	Sensitivity	Specificity	Precision
5	Logistic regression	71.1%	7.56 s	6.55%	89.89%	36.39%	72.31%
25	Logistic Regression	71.8%	9 s	6.22%	90.41%	37.34%	72.72%
50	SVM	71.3%	30 s	4%	93.83%	29.74%	71.16%

**Table 2:** Results obtained using holdout validation.

% of Data Held out for Validation	Performance Measures of Models						
	Best Performing Model	Accuracy	Training Time	% of False Negatives	Sensitivity	Specificity	Precision
5	Linear Discriminant	77.8%	9.7 s	0%	100%	37.5%	74.35%
15	SVM	70.4%	2.44 s	3.7%	94.25%	25%	70.08%
25	SVM	70.2%	1.53 s	2.66%	95.89%	22.78%	69.65%

**Table 3:** Performance of the 10 trained neural networks.

Hidden Layers	Neurons on Each Hidden Layer	Performance Measures of Networks			
		Number of Epochs	Performance (MSE)	Training Time [s]	Gradient
1	3	41	1.94	0 s	6.8
	10	26	1.2	0 s	10.7
	20	32	3.49	0 s	2.62
5	3	39	33.4	0 s	57.1
	10	16	2.15	0 s	6.07
	20	15	6.23 × 10^−4^	00:04 s	6.45
	40	16	5.29 × 10^−5^	62 s	2.73
10	3	56	2.9	0 s	25.5
	10	20	0.508	00:02 s	14.3
	20	14	5.9 × 10^−3^	00:17 s	3.89

## Data Availability

The data presented in this study are available on request from the corresponding author. The data are not publicly available due to ethical reason.
